# Evaluation of different media compositions promoting hepatocyte differentiation in the canine liver organoid model

**DOI:** 10.1016/j.heliyon.2024.e28420

**Published:** 2024-03-25

**Authors:** Vojtech Gabriel, Addison Lincoln, Christopher Zdyrski, Abigail Ralston, Hannah Wickham, Sydney Honold, Basant H. Ahmed, Karel Paukner, Ryan Feauto, Maria M. Merodio, Pablo Piñeyro, David Meyerholz, Karin Allenspach, Jonathan P. Mochel

**Affiliations:** a SMART Lab, Department of Biomedical Sciences, Iowa State University, Ames, IA, USA; b 3D Health Solutions Inc., Ames, IA, USA; c Laboratory for Atherosclerosis Research, Institute for Clinical and Experimental Medicine, Prague, CZ, Czech Republic; d Veterinary Diagnostic Laboratory, Iowa State University, Ames, IA, USA; e Department of Pathology, University of Iowa, Iowa City, IA, USA; f Precision One Health Initiative, Department of Pathology, University of Georgia College of Veterinary Medicine, 30602, Athens, GA, USA

## Abstract

Organoids are 3-dimensional (3D) self-assembled structures capable of replicating the microanatomy and physiology of the epithelial components of their organ of origin. Adult stem cell (ASC) derived organoids from the liver have previously been shown to differentiate into primarily mature cholangiocytes, and their partial differentiation into functional hepatocytes can be promoted using specific media compositions. While full morphological differentiation of mature hepatocytes from ASCs has not yet been reported for any species, the functional differentiation can be approximated using various media compositions.

Six differentiation media formulations from published studies on hepatic organoids were used for the differentiation protocol. Target species for these protocols were humans, mice, cats, and dogs, and encompassed various combinations and concentrations of four major hepatocyte media components: Bone morphogenetic protein 7 (BMP7), Fibroblast Growth Factor 19 (FGF19), Dexamethasone (Dex), and Gamma-Secretase Inhibitor IX (DAPT). Additionally, removing R-spondin from basic organoid media has previously been shown to drive the differentiation of ASC into mature hepatocytes. Differentiation media (N = 20) were designed to encompass combinations of the four major hepatocyte media components. The preferred differentiation of ASC-derived organoids from liver tissue into mature hepatocytes over cholangiocytes was confirmed by albumin production in the culture supernatant.

Out of the twenty media compositions tested, six media resulted in the production of the highest amounts of albumin in the supernatant of the organoids. The cell lines cultured using these six media were further characterized via histological staining, transmission electron microscopy, RNA *in situ* hybridization, analysis of gene expression patterns, immunofluorescence, and label-free proteomics. The results indicate that preferential hepatocyte maturation from canine ADC-derived organoids from liver tissue is mainly driven by Dexamethasone and DAPT components. FGF19 did not enhance organoid differentiation but improved cell culture survival. Furthermore, we confirm that removing R-spondin from the media is crucial for establishing mature hepatic organoid cultures.

## 1 Introduction

The liver is an essential organ that performs various metabolic functions, including producing circulating proteins, processing fat, proteins, and carbohydrates from food, storing glycogen, producing bile, metabolizing drugs, and many more. Mature hepatocytes are almost solely responsible for many of these metabolic processes. In the biopharmaceutical industry, hepatocyte cultures are used to investigate the metabolism and toxicity of potential drug candidates to gain approval from the Food and Drug Administration (FDA) [1].

In accordance with the principles of the 3Rs (*R*eplacement, *R*eduction, *R*efinement) [2], multiple cell lines have been established to study drug metabolism *in vitro*. Human-derived hepatic cell lines are a popular model for studying drug metabolism (for example HepG2, BC2, and HeparRG) [2], but these models typically fail to accurately replicate the *in vivo* hepatic microarchitecture, leading to poor cellular differentiation and lack of liver-specific functions [3]. For instance, cytochrome P450 enzymes, a family of enzymes responsible for drug biotransformation, were expressed at very low levels as compared to primary human hepatocytes, first reported by Wilkening et al. [4]. Furthermore, the expression of the constitutive androstane receptor (CAR), an inducer of P450 cytochromes, is undetectable in HepG2 cultures [5]. Similarly, the expression of the pregnane X receptor (PXR), another important xenosensor, is present at low or undetectable levels in HepG2 cultures [6].

Primary hepatocytes have also been extensively used to study *in vivo* drug metabolism, but the lifespan of these cells is limited and requires new donor donations for every experiment.

An alternative is taking advantage of the rapid progress in genetic engineering technologies, allowing the design of cells expressing drug metabolizing enzymes (for example cytochrome P450) [7]. Recent developments in the organoid technology field have enabled the investigation of stem cell-derived lines for drug metabolism testing purposes. Organoids serve as three-dimensional (3D) *in vitro* assemblies, faithfully modeling the microanatomy and physiology of the tissues from which they originate. One of the major advantages of 3D hepatic organoid cultures is their ability to represent all epithelial cell types found in adult liver tissue, ideally approximating the *in vivo* ratios of differentiated cholangiocytes to hepatocytes. Hepatic organoids have significant potential for applications in precision medicine, gene therapy, and regenerative medicine, as well as in fields such as pharmacology, microbiology, and basic research [8,9,10,11]. Induced Pluripotent Stem Cell (iPSC)-derived organoids offer a viable alternative to traditional two-dimensional (2D) cell cultures, addressing limitations such as restricted cell source and ethical concerns associated with Embryonic Stem Cell (ESC) lines. While iPSC-derived organoids can differentiate into tissues from any germ layer—unlike adult organoids, which are limited to endodermal differentiation [8]—they also have limitations in terms of lifespan and cytochrome P450 (CYP450) expression [12].

Adult Stem Cell (ASC)-derived organoids, first developed for intestinal tissues in 2009 by Sato et al. [13], have since extended to various research domains. Subsequent innovations include hepatic organoids derived from rodents [14], humans [15], canines [9], and felines [16]. Notably, canine hepatic organoids can be used to replicate human *in vivo* biology for translational research [4], especially in pharmacological studies of naturally occurring diseases [17]. One significant challenge lies in the complete differentiation of hepatic organoids into functional hepatocyte-like cells. Existing literature on hepatic organoids derived from human, rodent, and canine donors has noted a predominant presence of cholangiocytes, characterized by cytokeratin 19 (KRT-19) expression, especially in the initial growth stages [[9], [14], [15]]. This is in contrast to the *in vivo* hepatic tissue composition, where hepatocytes constitute approximately 70% of the cell population [18] and cholangiocytes make up only 3–5% [19]. The skewed phenotype towards cholangiocyte-like or ductal cell types in early hepatic organoid cultures is likely influenced by the initial growth factor composition established for ASC-derived intestinal organoids. Therefore, to achieve a more accurate *in vivo* representation of mature cholangiocytes and hepatocytes within hepatic organoids, it is imperative to investigate alternative media compositions that encourage ASCs from liver tissue to differentiate more effectively into mature hepatocytes.

Specialized differentiation protocols were developed to optimize the yield of hepatocytes in organoid cultures. These protocols use a combination of growth factors to facilitate stem cell expansion and differentiation, as well as biliary epithelial cell transdifferentiation via the Wnt/β-catenin signaling pathway [20]. Despite these advancements, the cells generated from these protocols have yet to fully display the functional characteristics of mature hepatocytes, such as albumin production, low-density lipoprotein (LDL) uptake, and glycogen storage comparable to *in vivo* conditions. Consequently, these cells are more aptly termed “hepatocyte-like cells” rather than fully mature hepatocytes [21]. While literature specifically focusing on hepatocyte differentiation media compositions is limited, six protocols have been described between 2013 and 2021 [14,15,16,[21], [22], [23]]. These protocols provide insights into the differentiation processes in adult stem cell-derived hepatic organoids from various species, including canines, felines, humans, and rodents.

**Fig. 1:**
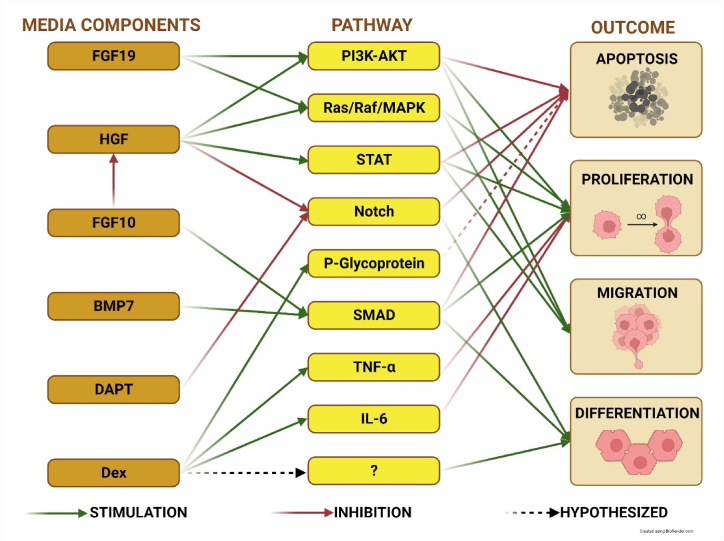
Liver organoid differentiation pathways. Summary of the most important media components for hepatocyte differentiation displaying the pathways and the overall outcome. Stimulation (green arrow), inhibition (red arrow), hypothesized interaction (dashed arrow), and unknown pathways (question mark) are denoted. FGF19 - Fibroblast Growth Factor 19; HGF - Hepatocyte Growth Factor; FGF10 - Fibroblast Growth Factor 10; BMP7 - Bone Morphogenetic Protein 7; DAPT - N-[N-(3,5-Difluorophenacetyl)-L-alanyl]-S-phenylglycine *t*-butyl ester; PI3K-AKT - Phosphatidylinositol-3-Kinase and Protein Kinase B pathway; Ras/Raf/MAPK - Ras/Raf/mitogen-activated protein kinase cascade; STAT - Signal Transducer and Activator of Transcription pathway; TNF-α - Tumor Necrosis Factor-alpha; IL-6 – Interleukin 6.

In this study, we subjected canine liver organoids to components of six of these protocols to assess their efficacy in inducing hepatic differentiation. Detailed attributes and compositions of these protocols are depicted in Fig. 1. The primary objective of this research was to evaluate the potential of various components of hepatocyte differentiation media in generating hepatocyte-like cells within canine hepatic organoids. We further characterized the cells cultured in the media that yielded the highest levels of canine albumin production, assessing them for specific morphological and functional markers indicative of mature hepatocyte differentiation.

## 2 Methods

### 2.1 Differentiation component selection

Six hepatocyte differentiation protocols that had previously been used for hepatic organoid cultures in four different species (human [[8], [24], [25]], rodent [12], dog [13], and cat [14]) were identified. The expansion and differentiation media composition was analyzed and compared to previously established protocols for the culture of canine organoids [15] (Table 1A). The canine organoid media previously published by our group [15] was supplemented by adding human recombinant hepatocyte growth factor (HGF) and fibroblast growth factor 10 (FGF10) [15], as these components were present in the expansion media of all six protocols available from the literature. Five additional media constituents from these protocols were additionally added: bone morphogenetic protein 7 (BMP7), fibroblast growth factor 19 (FGF19), dexamethasone (Dex), gamma-secretase inhibitor IX (DAPT), and activin-like kinase 5 inhibitor (A8301). Seven media components were removed: Rock inhibitor (ROCKi), Noggin, Nicotinamide (Nico), R-Spondin-1 (R-spo), GSKi, HGF, and FGF10 from the organoid expansion media (Table 1A, Table 1B). In our experimental design, the highest concentrations of any additional components cited in previous literature were employed. The differentiation process was initiated with the addition of Hepatocyte Growth Factor (HGF), Fibroblast Growth Factor 10 (FGF10), and Bone Morphogenetic Protein 7 (BMP7) where applicable. A secondary time point was set between Day 4 and Day 6 for the introduction of remaining components. To maintain experimental consistency, the addition of these subsequent components was uniformly executed on Day 5 post-initiation of organoid differentiation.

**Table 1A:**
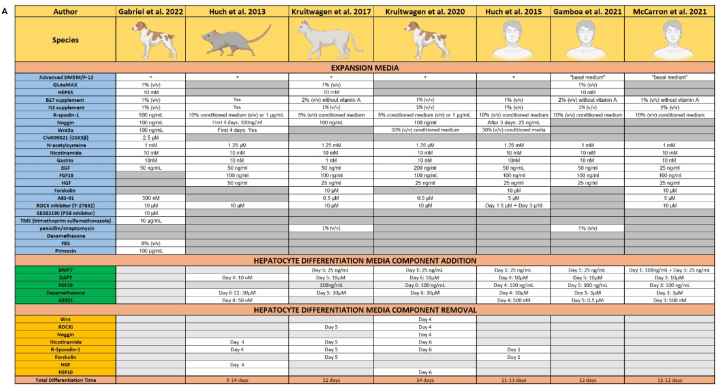
Culture media comparison. In this study, we compared six unique differentiation protocols from various species against our original media composition [26]. Both the composition of the expansion media and the total duration of the differentiation process were considered. Specific components were either introduced or removed to optimize the hepatocyte differentiation media.

Three components most frequently omitted from media compositions in the cited literature—R-spondin (R-spo) in four out of six protocols, Nicotinamide in three out of six protocols, and ROCK inhibitor in two out of six protocols—were selectively removed from our experimental media. Conversely, four components most commonly included in the media compositions from the literature—BMP7, DAPT, Dexamethasone (Dex), and FGF19—were chosen. These components were combined to create 20 unique differentiation media compositions for testing (Table 1B).

**Table 1B:**
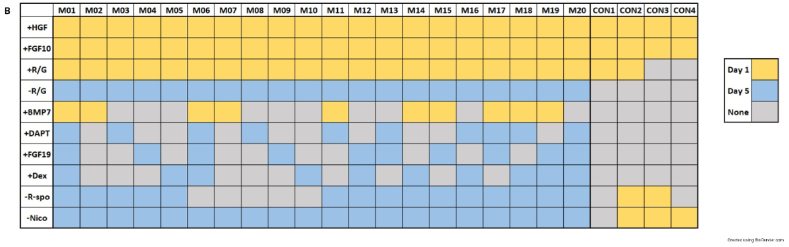
Culture media comparison. A total of twenty hepatocyte differentiation media and four control media were scrutinized in the initial experiment. Components were either added (such as HGF, FGF10, ROCK inhibitor/CHIR99021 (GSK3 inhibitor) referred to as R/G, BMP7, DAPT, FGF19, and Dex) or removed (like R/G, R-spondin, and Nicotinamide). The timing of these additions or removals is indicated by yellow (for Day 1) and blue (for Day 5) markers. Additionally, four control media were incorporated into the experimental design for comparative analysis. This framework allowed for a comprehensive evaluation of the effectiveness of various media compositions in promoting hepatocyte differentiation.

The hypothesized mechanisms of action for these added differentiation components are represented in Fig. 1. It is worth noting that Wnt withdrawal was intentionally excluded from these experiments, as prior studies have indicated that the removal of Wnt would lead to rapid deterioration of the cultures [9,27].

### 2.2 Cell count and albumin measurement

Hepatic organoid cell culture lines that had previously been derived from a healthy adult dog and were available in our bioarchive were thawed, passaged, and seeded in a 24-well plate at a density of ∼100,000 cells/well. Culture conditions for the growth and maintenance of canine organoids were used as previously described by our group [15]. Experiments were performed in triplicates. Twenty different media compositions were tested, with four organoid media:expansion media with ROCKi/CHIR99021 (GSK3 inhibitor) R/G; media with R/G without R-spo and Nicotidamide; media without R-spo and Nicotidamide; media without Nicotidamide serving as positive controls to ensure that the loss of these components will not hamper organoid growth.

The hepatocyte differentiation process was divided into two distinct phases. The first phase extended through the initial four days of culture and differentiation. This was followed by the second phase, which involved a change in media composition and lasted from Day 5 to Day 14, consistent with previously described protocols [26]. To assess the viability of organoid cultures in each media type, and to prevent the dissociation of organoid structures, the organoids were cultivated without undergoing cleaning or passaging—procedures elaborated in earlier publications. Culture supernatants were collected on Days 11 and 14 post-experiment initiation for the specific purpose of measuring albumin production, thereby serving as an indicator of the functional status of the hepatocyte-like cells within the organoids. On Day 14, cells were passaged, and their count and viability were evaluated for every well individually and in experimental duplicates using an automated cell counter (Countess II FL, Thermo Fisher) and trypan blue staining to assess cell viability, following a protocol published in 2015 [9]. Canine albumin concentrations were measured in the culture supernatant using a colorimetric ELISA immunoassay in triplicates (ab277078, Abcam; ELC-Albumin RayBiotech). Albumin production in the culture supernatant was measured following the manufacturer's instructions. Samples were analyzed on a microplate reader (BioRad iMark), and data were plotted with a four-parameter logistic curve using MyAssays Online (https://myassays.com) [16]. At this stage, the media yielding the highest concentrations of albumin in the supernatant (>50 ng/mL) were identified. Cut-off was set by diametrical differences when compared to other component combinations.

In subsequent experiments, these six media were used for the culture of organoid cell lines derived from two healthy adult dogs (1 M + 1F) in 12 replicates. Organoids were first defrosted and expanded in expansion medium as previously described [15,10]. They were then seeded at a lower density of ∼50,000 cells/well to decrease excessive apoptosis due to presumed overcrowding (a phenomenon described in a previously published protocol [15]). Organoids were finally cleaned during differentiation, as previously described [15]. Differentiated organoids were harvested for downstream analysis to characterize the phenotypic and functional differentiation status of the cells, including histopathology, morphological examination by bright field microscopy, transmission electron microscopy, RNAscope® for selected markers of hepatic differentiation (stem cell markers LGR5 and SOX9, and P450 enzymes CYP2B6 and CYP3A12), immunofluorescence for P-glycoprotein (P-gp) expression, as well as protein analysis of secreted markers in the culture supernatant using matrix-assisted laser desorption/ionization-time of flight (MALDI-TOF) mass spectrometry.

### 2.3 Label-free relative quantitative proteomics

The supernatant samples of culture M01 (representing the most commonly used hepatocyte differentiation composition from the literature) and the control were harvested and analyzed at the Iowa State University Office of Biotechnology Protein Facility. A label-free relative quantitative proteomics mass spectrometry method was performed to quantify the protein abundance ratio in the supernatant samples. The sequence of bovine serum albumin protein (BSA), which was abundantly present in the media, was searched using Mascot against Sprot-mammalia and with Sequest HT against Sprot-all [11]. The samples were searched with Mascot against Sprot-*Canis lupus familiaris* and Sequence HT against the PRTC sequences. Sample processing details and data analysis are available as Supplementary Material. The protein abundance ratio was used to identify the most differentially expressed proteins between organoids cultured in the differentiation media (BMP7, DAPT, FGF19, and Dex) and the organoids cultured in the control media.

### 2.4 Single-molecule RNA *in situ* hybridization

RNAscope® was utilized to perform RNA *in situ* hybridization experiments as previously described by Wang et al. [17]. In short, the following manufacturer-designed paired double-Z oligonucleotide probes against the canine target RNA were used: Cl-LRG5 (cat no. 405651, XM_846738.2, nt 517–1506), Cl-KRT7 (cat no. 838971, XM_005636798.2, nt 418–1640), Cl-CYP3A12 (cat no. 577821, NM_001003340.1, nt 2–1823) Cl-CYP2B6/CYP2B11 (cat no. 577831, NM_001006652.1, nt 121–1207) and Cl-Polr2a used as a positive control to confirm successful sample processing (cat no. 310981, XM_852751.3, nt 1846–2924). The RNAscope® 2.5 HD Reagent Kit-RED (cat no. 322350, Advanced Cell Diagnostics, Newark, CA) was used according to the manufacturer's instructions. FFPE sections were prepared based on the manufacturer's recommendations. Brightfield images (N > 10) were acquired on an Olympus BX43 microscope using a 40× objective. Fiji software was used for signal/cell area quantitative measurements [28] following the manufacturer's technical note [18].

### 2.5 Histology staining

For paraffin-embedding of the organoids, culture media was removed from the wells, and an FAA solution (Formalin-Acetic Acid-Alcohol) [15] was added to the samples still embedded in the extracellular membrane matrix (ECM; Matrigel for Organoids, Phenol Red-free Matrigel; Corning). After 24 h, FAA was removed, and 70% ethanol was added before samples were sent for embedding in traditional metal base molds and staining at the Veterinary Diagnostic Laboratory of Iowa State University. The slides were deparaffinized/hydrated using standard histological procedures. Specific staining protocols can be found in the Supplementary Material.

Staining included Hematoxylin & Eosin (H&E) for morphological evaluation by a board-certified pathologist. H&E-stained slides were provided to a histopathologist who was blinded to the identity of the samples to compare the percentage of canine organoid cells morphologically resembling cholangiocytes or hepatocytes, respectively, for each of the experimental media used. Periodic acid–Schiff (PAS) was used for detection of glycogen (red to magenta color), and PicroSirius Red for collagen I and III (red color) and cellular cytoplasm (yellow) detection. Furthermore, Masson's Trichrome was used to confirm presence of collagen I (blue), and periodic acid silver methenamine (PASM) was used for basal membrane identification. Additionally, Prussian Blue staining was used for identification of iron presence in the samples.

### 2.6 Transmission electron microscopy

Samples were placed in 1% paraformaldehyde, 3% glutaraldehyde in 0.1 M sodium cacodylate buffer, at pH 7.2 and fixed for 48 h at 4 °C. Samples were washed in cacodylate buffer 3 times for 10 min each, and post-fixed with 1% osmium tetroxide in 0.1 M sodium cacodylate buffer for 1 h at room temp. Samples were washed with deionized water 3 times for 15 min each, and en-bloc stained using 2% uranyl acetate in distilled water for 1 h. Samples were washed in distilled water for 10 min and dehydrated through a graded ethanol series (25, 50, 70, 85, 95, 100%) for 1 h. Samples were further dehydrated with 3 changes of pure acetone, 15 min each, and infiltrated with EmBed 812 formula (hard) for EPON epoxy resin (Electron Microscopy Sciences, Hatfield PA) with graded ratios of resin to acetone until fully infiltrated with pure epoxy resin (3:1, 1:1, 1:3, pure) for 6–12 h per step. Tissues were placed into beam capsules and were polymerized at 70C for 48 h. Thick sections (1.5 μm) were cut using a Leica UC6 ultramicrotome (Leica Microsystems, Buffalo Grove, IL) and stained with EMS Epoxy stain (a blend of toluidine blue-O and basic fuchsin). Thin sections were cut at 50 nm and collected onto single slot carbon film grids. TEM images were collected using a 200 kV JEOL JSM 2100 scanning transmission electron microscope (Japan Electron Optics Laboratories, USA, Peabody, MA) with a GATAN One View 4K camera (Gatan inc., Pleasanton, CA).

### 2.7 Immunofluorescence

Slide deparaffinization was achieved by washing in xylene twice for 10 min, followed by 100% ethanol wash twice for 1 min. Washes were performed with aggressive agitation. After deparaffinization was complete, the slides were allowed to air dry for 5 min and Heat-Induced Epitope Retrieval (HIER) was started with a Citrate buffer (pH 6) for 2 h at 75 °C using a HybEZ II Oven. This step was performed to remove any remaining bonds from the paraffin embedding process. After the HIER was complete, slides were allowed to cool to room temperature for 15 min and rinsed twice in phosphate-buffered saline (PBS) for 2 min each. One last wash was performed in PBS for 10 min and then the slides were permeabilized via incubation in 0.25% Triton in PBS for 20 min. Slides were rinsed in PBS three times to get any remaining Triton off them and then blocked for 1 h at room temperature in Casein in PBS (ThermoScientific, 37528). Once the blocking step was complete slides were allowed to sit overnight in p-glycoprotein (Invitrogen, PA5-33316) at 1:50 dilution in 4 °C. Once incubation was complete, samples were rinsed three times in PBS and incubated in donkey anti-rabbit secondary (Invitrogen, 2339822) at 1:1000 for 1 h. For nuclear staining, 4′,6-diamidino-2-phenylindole (DAPI) at 1:500 was added to the samples (Sigma, D9542-1 MG) for 20 min at room temperature. Slides were washed again in PBS for 10 min three times. A last rinse was performed with distilled water, and then slides were dried and mounted with Fluoroshield™ (Sigma, F6182-20 mL).

### 2.8 P-glycoprotein transport assay

Canine hepatic progenitor organoids were expanded using a pre-established protocol in traditional CMGF + R/G media, as described in earlier literature [26]. The experimental design included wells arranged in quadruplets: two containing differentiated organoids and two with non-differentiated organoids. The initial differentiation media comprised Nicotinamide (10 μM), R-spondin (0.5 μg/mL), Hepatocyte Growth Factor (HGF, 25 ng/mL), Fibroblast Growth Factor-10 (FGF-10, 100 ng/mL), and R/G. These components were supplemented in the media for the first five days of the experiment.

On Day 5, Dexamethasone and DAPT were introduced into the media, while R-spondin and Nicotinamide were withdrawn. By Day 14 of the differentiation process, Verapamil, a P-gp inhibitor, and Rhodamine, a substrate for the P-gp efflux protein, were added to the culture media. Specifically, the organoids (both M16 and control groups) were pre-treated with Verapamil (10 μM) for 1 h at a temperature of 37 °C. A single quadruplet of both non-differentiated and differentiated organoids was subjected to these inhibitory conditions. Subsequently, Rhodamine (100 μM) was added to each well and incubated at 37 °C for 5 min. The organoids were then washed three times with 500 μL of warm Dulbecco's Modified Eagle Medium (DMEM).

Imaging of the organoids was conducted 1-h post-treatment using a Keyence confocal immunofluorescence microscope [29]. Images were captured under each of the four conditions, focusing on the rhodamine fluorescence in the green channel.

### 2.9 Statistical analysis

Statistical outliers in cell counts were identified using the Robust Outlier detection based on the ROUT method with a stringent Q-value of 10%. Mean count data are reported along with their corresponding standard deviation (SD). Adjusted values for albumin concentration relative to cell number are also presented, accompanied by their respective SDs. A minimum of 10 representative images for each marker were captured using RNAscope® technology, and the signal area per cell signal area was calculated for each individual image. Outliers in these measurements were identified using the same ROUT method with a Q-value of 10%. Data normality was assessed using the D'Agostino & Pearson test. Comparisons between different media compositions were performed using ordinary one-way analysis of variance (ANOVA). *GraphPad Prism 9 was used to perform statistical evaluation.* Statistical significance was set at *P*-values <0.05).

## 3 Results

### 3.1 Organoid morphology

**Fig. 2:**
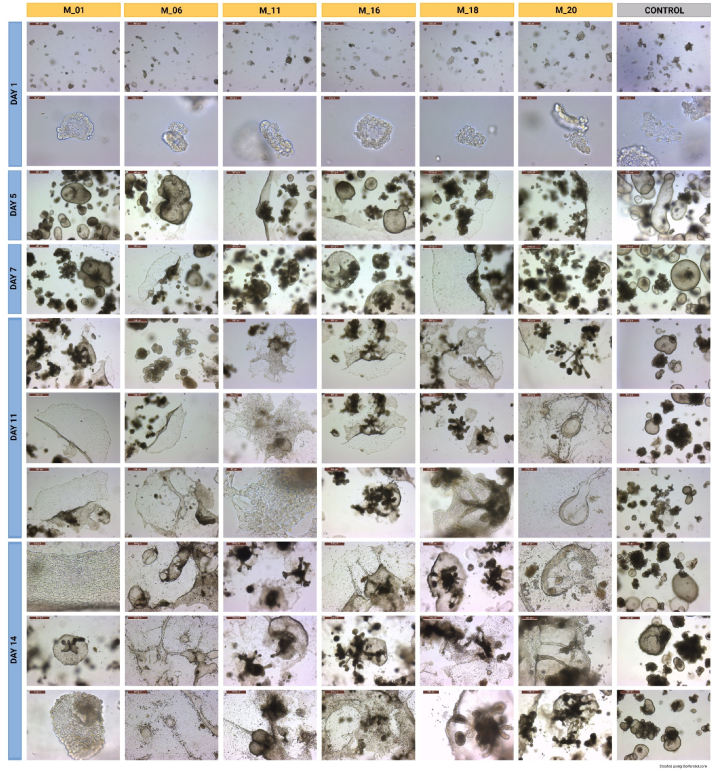
Organoid morphological assessment – Images of treatment groups and control captured on days 1, 5, 7, 11, and 14 via light microscopy. The scale bars are in μm. Abundance of dark/black areas are suggestive of cell death.

Distinct differences in organoid morphology were identified during hepatocyte differentiation using different media compositions; a summary of morphologies can be seen in Fig. 2. Spheroids formed and rapidly expanded in size on the second day of the third passage. By Day 5 of culture, all of the experimental media used resulted in an altered morphology of the organoids compared to the control media, including monolayer-like growth around the extracellular basement membrane drop (a phenomenon previously described as epithelial-to-mesenchymal transition [19]). While the control group treated with organoid expansion media alone resulted in the expansion of mostly spheroids and only a few more differentiated, budding structures, the organoid cell lines cultured in the experimental media differentiated mainly into budding structures with only a few spheroids. Apoptotic changes in the organoids were present in all experimental samples by Day 7 of culture, as described in a previously published protocol [15]. This change was predominantly seen in the center of the extracellular basement membrane drop, while organoids on the outside of the extracellular membrane matrix seemed more proliferative and less apoptotic as seen in Fig. 2. Structures that partially transdifferentiated into the monolayer plaques showed no signs of apoptosis. By Day 7, experimental and control samples contained a high density of medium-to very-large organoids based on scoring previously described in a standardized protocol [15].

Interestingly, the organoids in M16 showed entirely different morphologies, with groups of traditional budding organoids forming larger common spheroid megastructures (Fig. 2). These organoids transitioned into fibroblast/mesenchymal-resembling cells and formed a monolayer expanding into a halo around the basement membrane drop by Day 11. Organoid samples of the control group (CMGF + R/G) did not change in morphology but began to undergo apoptosis by Day 11, likely due to the lack of passaging and overcrowding, which was expected due to the experimental design. All organoids in experimental samples suffered complete apoptosis of cells in the central section of the Matrigel drop but created interconnected structures closer to the drop border, changing into cellular monolayers and escaping out of the ECM border (Fig. 2, Day 14). M01 resulted in the highest organoid proliferation rate with the least amount of apoptosis of organoids out of all experimental samples, as assessed by cell count using trypan blue (265,000 ± 32,527 cells/well) and observation of the cells via brightfield microscopy. By Day 14, M01-cultured organoids created massive cellular 2D plaques and distinct organoids consisting of cell clusters without a lumen (Fig. 2, Day 14). Cells cultured in M06 expanded into a monolayer-like structure with a set of interconnected tubular structures with chambers. M11, M16, M18, and M20 created distinct types of organoids characterized by tubular growth out of the organoid center and expansion from the outer edge of the organoid structures (Fig. 2, Day 14). M20 seemed to express the most elaborate structures of interconnected tubules and chambers out of all media tested (Fig. 2, Day 14). Apoptosis occurred on Day 14 in all samples, including control, due to the lack of organoid passaging and regular cleaning.

### 3.2 Albumin production

**Fig. 3:**
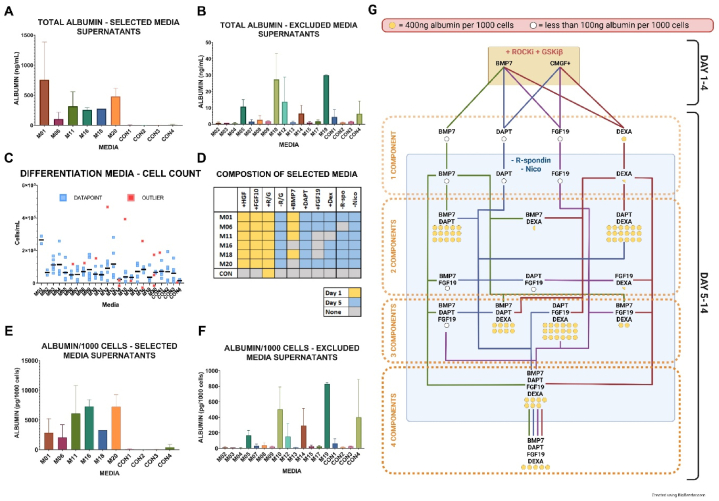
Albumin production. 3A - The media compositions that resulted in the highest levels of albumin production, as measured by ELISA on Day 14 of differentiation, were selected for the final experiment. 3B - Media that yielded low levels of albumin were excluded from further analysis. 3C - Cell counts from the initial experiment are presented, with the experiment being conducted in triplicates and measurements taken in duplicates. Data points are represented in blue, and outliers are highlighted in red. 3D - Detailed compositions of the six media types chosen for further characterization are provided. 3E - Characterization of supernatants from the selected media is outlined. 3F - Albumin concentrations in the “excluded media” are normalized against cell count for comparative analysis. 3G - A summary of the albumin measurement experiment, involving 20 different media, is presented. The data are categorized based on individual components and their combinations. Media that yielded less than 100 ng of albumin per 1,000 cells are indicated by a white circle, and those yielding 400 ng of albumin per 1,000 cells are indicated by a yellow circle. Samples in which R-spondin was removed on Day 5 are marked with blue squares. Media that were supplemented with R-spondin throughout the duration of the experiment are represented outside the rectangle. This structured presentation allows for a nuanced understanding of how different media compositions and their components influence albumin production and cell differentiation. It forms the basis for further optimization and in-depth analysis.

The highest average albumin yield among the 20 different media compositions was recorded in M01, yielding 757 ± 625 ng/mL, followed by M20 (480 ± 135 ng/mL), M11 (316 ± 243 ng/mL), M18 (273 ng/mL), M16 (254 ± 40 ng/mL), and M06 (104 ± 110 ng/mL) (Table 1A, Table 1BA and B). Notably, M18 was not measured in triplicates due to the loss of organoid cells during sample processing. A threshold of 100 ng/mL in albumin concentration was established for inclusion in the final experiment. The mean concentrations of albumin in the supernatant for the other two media compositions ranged from 30 ± 54 ng/mL in M19 to 0.5 ± 0.4 ng/mL in M04. Concurrently, albumin concentrations in control media ranged between 6.3 ± 7.8 ng/mL and 0.9 ± 0.4 ng/mL. The average albumin concentration for the final experimental media was 387 ± 351 ng/mL, in contrast to the control media, which had an average of 2.8 ± 3.6 ng/mL, and the excluded media with an average of 6.7 ± 10.3 ng/mL. These results on albumin production are summarized in Fig. 3E and F.

Cell counts for differentiated and control samples, as well as the results of albumin measurements in the supernatant for each media composition, are elaborated in Fig. 3C and D and Supplementary material. Briefly, the initial experiment assessing albumin production by organoids across 20 differentiation media was conducted in triplicates, while cell counts were performed in duplicates, totaling six measurements. These cell counts served to normalize the albumin values, with the highest cell count observed in M01 at 265,000 ± 32,527 live cells per well. The cell counts for other selected media were as follows: M06 (51,140 ± 15,783 cells/well), M11 (51,800 ± 30,270 cells/well), M16 (34,960 ± 9,038 cells/well), M18 (83,850 ± 15,433 cells/well), and M20 (66,467 ± 5,948 cells/well). For control samples, cell counts ranged from 15,932 ± 4,083 cells/well in CON4 (expansion media w/o Nico) to 72,480 ± 40,773 cells/well in CON1 (expansion media + R/G).

Albumin concentrations in the supernatant were normalized to the live cell count per well using the trypan blue exclusion method, as previously described [30]. In this regard, M16 exhibited the highest albumin production, averaging 7,271 ± 1,137 pg/mL/1,000 cells, followed by M20 (7,215 ± 2,024 pg/mL/1,000 cells), M11 (6,094 ± 4,701 pg/mL/1,000 cells), M18 (3,256 pg/mL/1,000 cells), M01 (2,857 ± 2,358 pg/mL/1,000 cells), and M06 (2,037 ± 2,164 pg/mL/1,000 cells). The control media exhibited albumin concentrations in the supernatant that varied between 13.54 ± 5.5 pg/mL/1,000 cells in CON2 and 399.1 ± 489.2 pg/mL/1,000 cells in CON4. When the data were normalized against cell counts, the mean albumin concentrations for the selected media, control media, and excluded media were 5,030 ± 3,161 pg/mL/1,000 cells, 97 ± 216 pg/mL/1,000 cells, and 133 ± 229 pg/mL/1,000 cells, respectively. For comparative purposes, the differentiation media in Huch's human hepatic organoid media protocol [15] yielded approximately 350 pg ALB/day/1,000 cells.

### 3.3 RNAscope®

**Fig. 4:**
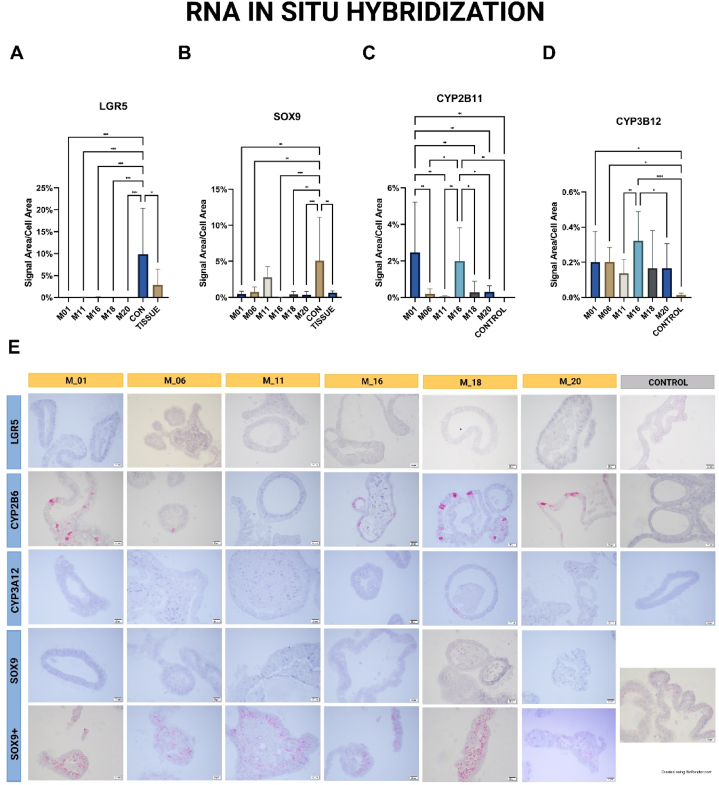
RNA *in situ* hybridization. RNA *in situ* hybridization **4A** - stem cell marker LGR5; **4B** – ductal/cholangiocyte cell marker SOX9; **4C** – hepatocyte functional marker CYP2B11; **4D** – hepatocyte functional marker CYP3A12; **4E** - Representative pictures of slides created via RNAscope®. A positive signal (red) indicates the individual mRNA of the marker. Structures with lower expression (SOX9) and higher expression (SOX9+) were evident in all experimental cultures.

RNA *in situ* hybridization assays were conducted for canine-specific markers LGR5, CYP2B11, CYP3A12, and SOX9, as depicted in Fig. 4E. Expression levels were quantified as the ratio of signal area to cell area percentage, in accordance with the manufacturer's guidelines. For LGR5 mRNA, expression levels in organoids cultured in different differentiation media ranged from 0.003 ± 0.004% to 0.057 ± 0.031% in M11 (Fig. 4A). In contrast, the control group displayed an average expression of 9.846 ± 10.5%. In donor hepatic tissue, LGR5 expression as assessed by RNAscope® was 2.856 ± 3.631%.

SOX9 expression levels were found to average 2.773 ± 1.478% in M11, while the control group averaged 5.07 ± 6.048%. Expression levels for SOX9 in other differentiation media ranged from 0.023 ± 0.019% in M16 to 0.742 ± 0.683% in M06 (Fig. 4B).

For CYP2B11, the highest expression levels were observed in M01 (2.462 ± 2.757%) and M16 (1.998 ± 1.821%). In other differentiation media, CYP2B11 expression was considerably lower, ranging from 0.052 ± 0.052% in M11 to 0.302 ± 0.333% in M20. Notably, CYP2B11 was not expressed in the control group (Fig. 4C).

Lastly, CYP3A12 expression was most pronounced in M16, registering at 0.322 ± 0.167%, while the control group exhibited the lowest expression at 0.013 ± 0.011%. Expression levels for CYP3A12 in other differentiation media were found to range from 0.137 ± 0.080% in M11 to 0.202 ± 0.082% in M06 (Fig. 4D).

### 3.4 Histology staining

**Fig. 5:**
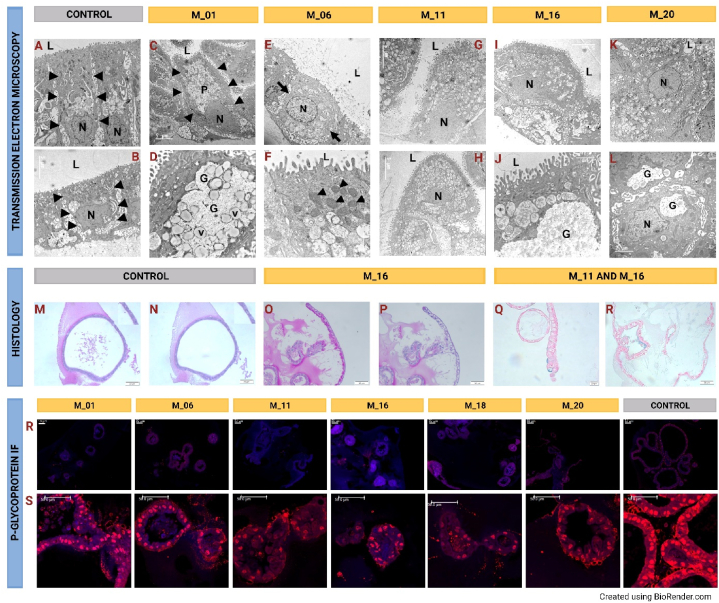
Microanatomical evaluation. **5A**, **5B** – Example of undifferentiated cells. These cells were polarized and columnar (left, arrowheads) to cuboidal (right, arrowhead) with irregular nuclei (N) mostly in the basolateral cytoplasm. **5C**, **5D** - Example of M01 cells. Polarized (left, arrowheads) cells had nuclei (N) in the basolateral cytoplasm. Note the lumen (L) and electron lucent (pallor, P) region in the cytoplasm. The electron lucent regions contained granular material in a localized region of electron lucent consistent with glycogen (G) with multiple homogenous vacuoles (v). **5E**, **5F** – Example of M06 cells. Polarized cuboidal cell (left, arrows) with cuboidal round, centrally oriented nuclei (N) in the cytoplasm. Note the lumen (L) and aggregates of mitochondria (arrowheads right) prominent in some cells. **5G**, **5H** - Example of M11 cells. Polarized columnar (left) to cuboidal cell (right) with basolateral nuclei (N) in the cytoplasm. **5I**, **5J** - Example of M11 cells. Polarized columnar (left) to cuboidal cell (right) with basolateral nuclei (N) in the cytoplasm. Note the lumen (L). **5K**, **5L** - Example of M16 cells. Polarized to low cuboidal cell (left) with basolateral nuclei (N, left) in the cytoplasm. Note the lumen (L) and also an electron lucent region of glycogen (G, right). **5M**, **5N** - Example of dPAS in undifferentiated controls. Cells have similar nominal differences suggesting basal glycogen levels (see insets). **5O**, **5P** - Example of M01 cells with PAS and dPAS stains (left and right respectively). Note the magenta coloration in light-colored cells on the left (inset) and the lack of staining if glycogen is removed prior to staining (dPAS stain). The lack of staining on the right and the pallor in the cells are indicative of glycogen and water accumulation in the cells. **5Q**, **5R** - Example of M11 and 16 cells. Note the rare AB + staining for mucus. 5R, 5S – P-glycoprotein detected via IF captured at 20× resolution and 60×.

**Fig. 6:**
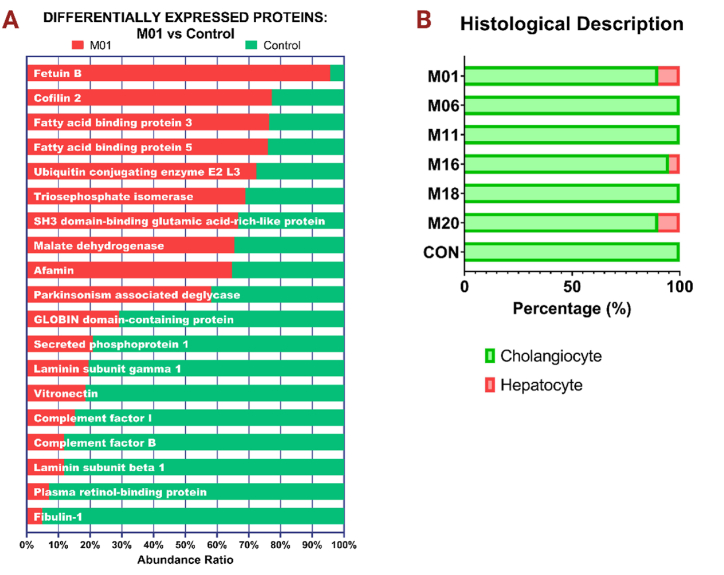
Proteomics and morphological description. **6A** - Comparison of M01 and control supernatant based on proteins with highest and lowest abundance ratios acquired using label-free relative quantitative proteomics technique. 6B - the histological ratio of cholangiocytes vs. mature hepatocytes in tested media as described by a board-certified pathologist.

Organoids were stained with H&E, PAS, PicroSirius Red Stain, Masson's trichrome, PASM, and Prussian Blue. Fig. 5M-R offers representative images. H&E staining was used to describe general morphology characteristics. More than 90% of cells were identified as cholangiocytes by a board-certified veterinary pathologist (DM) in organoids cultured in all of the experimental media conditions, while the control group was almost entirely composed of cells resembling cholangiocytes. Other samples contained up to 10% of differentiated hepatocytes, including M01 (10%), M16 (5%), and M20 (10%) (Fig. 6B). Hepatocytes were identified by the same pathologist as polyhedral cells with acidophilic cytoplasm that contained dotted basophilic regions representing rough endoplasmic reticulum and ribosomes. The center of the cells contained between two and four large and spherical nuclei containing at least two nucleoli. Cholangiocytes were identified as variable in shape from cuboidal to columnar and having less dense eosinophilic cytoplasm and containing a single central dense basophilic nucleus with a single nucleolus. The apical membrane aspect of these cholangiocyte-like cells caved in, and in some cases, microvilli were observed. Most cells expressed ballooning degeneration and accumulation of lipid droplets.

Masson's trichrome was used to stain for collagen I and showed more hepatocyte background (red) in the tested media than in control media cultured organoids. Collagen is primarily produced by hepatocytes and fat-storing cells [20]. Collagen was detected in cellular monolayers and in a cellular apoptotic debris caused by shedding in the lumen of organoids. Cells cultured in M16 expressed a lower number of cells with ballooning degeneration than controls, while cells cultured in M25 expressed high collagen production combined with a high degree of ballooning degeneration. Control group organoids (cultured in M25) did not express any ballooning degeneration of cells. PAS staining to identify basement membrane was not found in any organoids examined but was found in tissue samples, indicating the absence of basement membrane structures in the organoid cultures. Prussian Blue staining was performed to detect excessive iron accumulation and was not found to be present in either the organoids or the original tissue. Picrosirius red was much more highly positive for collagen staining in differentiation media-treated organoids than in controls. PAS staining was used for detection of glycogen and was not identified in any of the slides.

### 3.5 Label-free relative quantitative proteomics

Proteomic analysis was employed to identify and quantify additional proteins specifically in one selected media, M01, and these findings were compared with those from the supernatants of control cultures. The abundance of proteins in the supernatant for M01 was scrutinized and subsequently correlated with the control sample, with the results being expressed as a ratio of protein abundance to cell number. The choice of M01 for this analysis was deliberate, as it represents a combination of components that is traditionally utilized in hepatic differentiation protocols [15,16,[21], [22], [23]]. Differentially expressed proteins between the two media were defined as more than 10% difference in abundance of specific proteins between M01 and control media, and included Fetuin B (FETUB), Cofilin 2 (CFL2), Fatty acid binding protein 3 (FABP3), Fatty acid binding protein 5 (FABP5), Ubiquitin-conjugating enzyme E2 L3 (UBE2L3), Triosephosphate isomerase (TPI), SH3 domain-binding glutamic acid-rich-like protein (SH3BGRL), Malate dehydrogenase (MDH1), and Afamin (AFM). Most differentially expressed proteins in the controls compared to the M01 treated sample included Fibulin-1 (FBLN1), Plasma retinol-binding protein (RBP), Laminin subunit beta 1 (LAMB1), Complement factor B (CFB), Complement factor I (CFI), Vitronectin (VTN), Laminin subunit gamma 1 (LAMC1), Secreted phosphoprotein 1 (SPP1), GLOBIN domain-containing protein, and Parkinsonism associated deglycase (PARK7). Key proteomic data are summarized in Fig. 6A.

### 3.6 Transmission electron microscopy

Transmission electron microscopy (TEM) images are presented in Fig. 5A–L for visual assessment. Upon comparison with the donor tissue, it becomes evident that the organoids have not achieved full differentiation into morphologically mature hepatocytes. While the organoid cells overall sustained their cholangiocytes-like morphology, several markers of some degree of hepatocyte differentiation were also observed, including redundant mitochondria that are normally found in hepatocytes, the presence of secretory granules in the cells which is reminiscent of hepatocytes producing bile, and the formation of bile canaliculi of adjacent hepatocytes.

### 3.7 P-glycoprotein immunofluorescence and transport assay

**Fig. 7:**
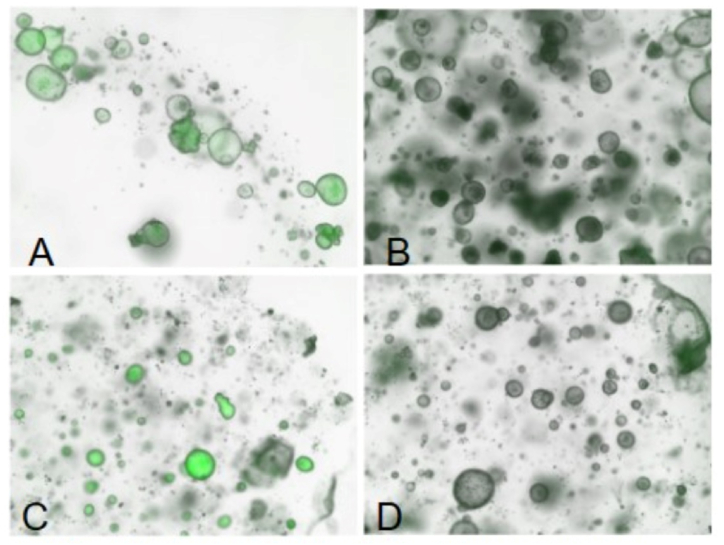
P-Glycoprotein verapamil/rhodamine transport assay. **7A** - In the control group, undifferentiated canine hepatic organoids were exposed to rhodamine. The accumulation of rhodamine in the lumen of the organoids confirmed the functional expression of the P-glycoprotein (P-gp) efflux protein. **7B** - Upon the introduction of verapamil, a known P-gp inhibitor, into the media, the intraluminal accumulation of rhodamine within the organoids was notably inhibited. This suggests the role of P-gp in the initial rhodamine accumulation. **7C** – In a repeat experiment employing differentiation media M16, similar intraluminal accumulation of rhodamine was observed, again implying functional activity of the P-gp transport protein. **7D** – Similar to 7B, the addition of verapamil to the culture media inhibited the P-gp-mediated efflux of rhodamine.

As illustrated in Fig. 5R and S, the control group displayed a noticeably higher expression of P-gp compared to all other experimental groups. This elevated expression in the control group was anticipated, given the well-documented abundance of P-gp in cholangiocytes [31]. Rhodamine uptake was observed in organoids cultured in both control and differentiation media, except when the media contained Verapamil. This observation corroborates the abundant expression of P-gp in the cells of the organoid cultures, particularly in the M16 and control groups. Upon the introduction of Verapamil, rhodamine uptake by the organoids was effectively inhibited within an hour. Representative images showcasing these experimental outcomes are presented in Fig. 7A–D. P-glycoprotein seems to be present mainly in the nucleoplasm. We are unsure if this is caused by the method of stem cell expansion. It seems this situation is not unique, though, and P-gp is localized both in the nucleoplasm and the plasma membrane, as seen in the images.

## 4 Discussion

Hepatic organoids derived from ASCs sourced from liver tissues across various species have been previously characterized as being predominantly composed of cholangiocytes or ductal cells [[9], [14], [15]]. This stands in stark contrast to the *in vivo* biology in liver tissue, where mature hepatocytes are the dominant cell type, unlike other organoid cell lines, such as enteroids, where the *in vitro* ratio of differentiated cells more closely approximates the *in vivo* situation. Since mature hepatocytes play a pivotal role in the metabolism of drugs, it is critical to establish robust culture protocols aimed at promoting the differentiation of liver-derived ASCs into hepatocyte-like cells [10]. In this study, we investigated 24 unique combinations of media compositions and assessed the resulting organoid cultures for characteristic morphological features and expression patterns pertinent to hepatic stem cells, mature hepatocytes, and mature cholangiocytes. Additionally, we evaluated the functional capabilities of these organoid cell lines in terms of albumin production and the expression of critical drug transporters and metabolic enzymes integral to mature hepatocyte function [15]. For each species previously described in the literature—namely mouse [14], cat [16], dog [21], and human [15]—one hepatic organoid differentiation protocol was identified. Moreover, two novel media compositions were formulated based on the most recent advancements in human hepatic organoid media composition [22,23]. Our laboratory had previously published a canine organoid differentiation media [26], which was enriched with Hepatocyte Growth Factor (HGF) and Fibroblast Growth Factor 10 (FGF10) across all the experimental protocols reported herein. FGF10 is a protein of the fibroblast growth factor family that plays an important role in different stages of epithelial cell morphogenesis and cellular differentiation [21].

HGF, also called scatter factor or hepatopoietin A, is a potent mitogen for hepatocytes mainly secreted by mesenchymal cells [22]. HGF can activate the Phosphatidylinositol-3-Kinase and Protein Kinase B (PI3K-AKT) pathway promoting hepatic oval cells (which are hypothesized to exhibit hepatic stem cell progeny) proliferation [23] and migration [26], while also resulting in an anti-apoptotic effect [27]. Finally, HGF is also known to downregulate the Notch signaling pathway in fetal liver stem cell cultures, a key mechanism for the successful differentiation of hepatocytes *in vitro* [30]. HGF was eventually withdrawn in Huch's original murine protocol [12], but this particular combination of factors was not investigated in our study as it was absent from all other published protocols [[8], [24], [25],^13^,^14^].

Overall, four major components were added to the differentiation media: BMP7 in 5/6 protocols, FGF19 in 5/6 protocols, and DAPT and Dex in all protocols. Specific media constituents were removed from the media to allow differentiation into functional hepatocytes. Among those, ROCKi was removed from 2/6 protocols, Nicotinamide from 3/6 protocols, and R-spondin-1 from 4/6 protocols. Partial differentiation into hepatocyte-like cells typically occurred on Day 3–6, with complete differentiation established for all culture conditions by Day 14.

Organoid morphology was evaluated using bright-field microscopy, revealing significant differences between the experimental media and the control group. Specifically, the experimental media led to an atypical morphological pattern. Unlike the conventional spheroids and budding organoids commonly seen in most epithelial ASC-derived mammalian organoid cultures, the experimental media induced a more interconnected pattern featuring epithelial-to-mesenchymal transition (EMT) around the extracellular basement membrane border. Previous studies have reported instances of stagnant organoid expansion [9], a phenomenon that was also observed in our research. During Days 7–14 of culture, we noted the occurrence of organoid apoptosis centered around the basement membrane. The outer cells appeared to undergo a transition from an epithelial to a more mesenchymal phenotype, and these mesenchymal-like cells continued to expand. This complex morphological shift has been previously linked to tissue repair processes, particularly in the context of tissue fibrosis, but remains poorly understood [32].

The concentration of canine albumin in the culture supernatant served as a screening tool to gauge the functionality of mature hepatocytes. Six media compositions, each yielding an average albumin production greater than 100 ng/mL, were selected for further investigation. The components commonly found in these high albumin-yielding media included DAPT in all six media, Dex in five out of six, BMP7 in four out of six, and FGF19 in three out of six. In stark contrast, the albumin yield in the four control media was between 15 and 111 times lower than the lower limit cutoff value established for the six media chosen for additional study. Subsequent analyses were conducted to further determine the influence of each media component (DAPT, BMP7, FGF19, Dex) on albumin production. Interestingly, individual components appeared to exert limited impact on canine albumin production when used in isolation. This observation was consistent across media both containing and lacking R-spondin. This data suggest that the synergistic effect of multiple components likely plays a crucial role in influencing hepatocyte functionality, rather than the action of individual components. While higher production of albumin by functionally active hepatocytes was observed when using 2-, 3-, and 4- component media, these optimized culture conditions did not reach production levels reported in primary hepatocyte cultures [33].

N-[N-(3,5-Difluorophenacetyl)-L-alanyl]-S-phenylglycine *t*-butyl ester (DAPT) acts as a γ-secretase inhibitor and an indirect Notch inhibitor [34]. DAPT was previously described to decrease epithelial stem cell survival and promote differentiation in a murine incisor epithelial stem cell model [35], as well as an *in vivo* rodent model of hepatocellular carcinoma [36]. DAPT further prevented hepatocyte apoptosis in a murine model of liver fibrosis [37] and nonalcoholic fatty liver disease HepG2 2D cell line [38].

The primary effect of DAPT appears to be associated with the inhibition of Notch signaling [39], which in turn exerts an anti-proliferative and pro-differentiation effect on hepatocytes. This effect has also been previously documented in a murine model of leukemia [40]. Wang et al. confirmed the critical role of DAPT in promoting hepatocyte differentiation when they treated fetal liver stem/progenitor cells with this compound in 2012 [41]. In our study, DAPT emerged as an important driver of hepatocyte differentiation, particularly when it was combined with BMP7 and Dex. However, this pro-differentiation effect was not observed when DAPT was combined with FGF19, indicating that the effect of DAPT on hepatocyte differentiation may be context-dependent, potentially requiring specific synergistic interactions with other factors for optimal effectiveness. A combination of DAPT and Dex resulted in the highest albumin-yielding medium, with the addition of FGF19 not improving the albumin yield any further [29]. FGF19 on the other hand prevented apoptosis [42].

Addition of Fibroblast Growth Factor 19 (FGF19) in the media for primary hepatocyte culture has previously been described to decrease albumin yield when added to a combination of BMP7 and DAPT [43]. FGF19 is a ligand of FGF Receptor 4 (FGFR4), a mediator of hepatocyte proliferation, and suppresses primary bile acid synthesis [31].

The combination of all four components yielded significant albumin concentrations of 757 ± 624 ng/mL in the supernatant of our organoid cell lines when R-spondin was simultaneously removed from the culture. However, the albumin concentration achieved in the supernatant using this protocol was still lower than when BMP7 with DAPT, or when DAPT with Dex were combined altogether. These data suggest a neutral to negative effect of FGF19 addition on enhancing hepatocyte differentiation in canine hepatic organoids. Our results also indicate that DAPT may play a key role in hepatocyte differentiation of canine organoid cultures. DAPT did not enhance differentiation when used by itself, however, differentiation was achieved when DAPT was combined with BMP7 and Dex. It is worth mentioning that a low cell count was observed in both the differentiation and control samples following the experiment. This outcome is likely attributable to inadequate organoid maintenance, a consequence of the experimental conditions employed [26]. Interestingly, this phenomenon was not observed in the organoid culture treated with M01, which experienced a remarkable 5-fold expansion during its 14-day differentiation period. This suggests that the M01 media composition may offer certain advantages in supporting organoid proliferation and maintenance, a finding that warrants further investigation.

Hepatocyte-derived plasma proteins were the main products identified in the M01 culture media (e.g., FETUB, CFL2, and AFM) in our study. According to data from the Human Protein Atlas [44], these proteins are most likely derived from functionally mature hepatocytes. FABP5 has been previously described to induce ETM transition of cells in hepatocellular carcinoma (HCC), a process we have observed to take place in our “selected media” cultures [45]. FABP3 was previously described to be upregulated in the liver tissue hepatic steatosis zebrafish model [46]. Furthermore, UBE2L3, which was previously shown to be involved in hepatocyte proliferation and migration in HCC [47], was more abundant in the differentiation media culture. TPI is a protein abundantly found in hepatocyte differentiation media, and has been shown to have anti-proliferative effects on HCC tumor cells [48], including suppression of growth, migration, and invasion of tumor cells. MDH1, which functions as part of the tricarboxylic acid cycle, is highly expressed in hepatocytes due to the abundance of mitochondria found in these cells [49]. SH3BGRL3 seems to be more abundantly expressed in differentiated cholangiocytes than mature hepatocytes, and FABP3 is altogether not very commonly expressed in the liver [50]. However this result could be interpreted as a false positive finding due to difficulties in canine protein sequence identification when compared to human or murine libraries that are available to date. Afamin, a plasma protein derived from hepatocytes [51], was identified in our cultures, further supporting the presence of functional hepatocytes. Additionally, Parkinsonism-associated deglycase (DJ-1), known to be upregulated in hepatocellular carcinoma (HCC), was also detected. This protein could potentially serve as a negative prognostic marker for HCC [52]. Collectively, the proteomics data indicates that the experimental media facilitated partial differentiation into functional hepatocyte-like cells, providing a promising avenue for further research into optimizing differentiation protocols.

Proteins that were found to be enriched in the supernatant of the control group cultures as compared to M01-treated organoids included Fibulin-1, with an abundance ratio of 20 times higher than in control cultures. According to the Human Protein Atlas [44], cholangiocytes preferentially express this glycoprotein which functions to stabilize extracellular matrix, while Fibulin-1 expression has not previously been reported in canine differentiated hepatocytes [53]. SPP1 is a marker of ductal cells and is commonly expressed in cholangiocytes [54]. LAMC1, an extracellular matrix component typically expressed by cholangiocytes and other molecules of the laminin family, has been previously shown to drive cholangiocyte differentiation [55]. VNT, CFI, LAMB1, CFB, and RBP are expressed in both hepatocytes and cholangiocytes [44]. The elevated expression of specific proteins in the control group of our experiments may be attributable to a higher concentration of stem cells and hepatic progenitor cells in our cultures compared to what is typically observed in adult liver tissue. This hypothesis is supported by the elevated levels of LGR5 and SOX9 expression, as measured by RNAscope® in our experiments. The presence of these markers suggests a stem cell-rich environment, which could account for the differential protein expression observed.

CYP2B11 is a homolog of human CYP2B6, and its expression is several times higher in canine liver as compared to the human liver [56]. It serves as a marker of hepatocyte function [57]. Gene expression of CYP2B11 was statistically significantly different in the organoids treated with combination media of all components (M01) and the media composition containing DAPT + Dex (M16) as compared to the control media-treated cell lines. CYP3A12 is another important marker of hepatocyte function in dogs [57] and tended to be more highly expressed in all of the organoid cell lines incubated with experimental media as compared to control conditions, with the combination of DAPT and Dex (M16) being superior to all other differentiation medias in stimulating CYP3A12 expression. LGR5 is a marker expressed by undifferentiated epithelial stem cells and, as expected, expression of this markers was significantly lower in all differentiation media as compared to control. Finally, SOX9 represents a marker of cholangiocytes/ductal precursor cells, and most of the experimental media-treated organoids in our study expressed significantly lower levels of this marker when compared to control. Taken together, the expression levels of stem cell and cholangiocyte-specific markers were reduced in all our experimental media compared to the controls. This suggests significant progress in the differentiation towards mature hepatocytes when utilizing these experimental media. Additionally, the findings point to a pivotal role for the inclusion of Dex in driving hepatocyte differentiation in organoid cultures.

Morphologically, most cells in our experimental cultures were described as phenotypically resembling cholangiocytes, except for the cultures derived from incubation with M01 and M20 media (with 10% mature hepatocytes) and M16 (with 5% mature hepatocytes). While the majority of cells in our canine hepatic organoid cultures exhibited morphology typical of cholangiocytes, significant expression of hepatocyte-specific markers was also noted. This observation aligns with previous findings [9] and underscores the challenges associated with achieving proper cellular morphology during hepatocyte differentiation in culture. This difficulty is one reason why the term “hepatocyte-like cells” is frequently employed in the literature [58]. Functional hepatocyte differentiation in our experimental cultures was confirmed through transmission electron microscopy, revealing characteristic features such as an abundance of mitochondria, the presence of secretory vacuoles, and the formation of bile canaliculi between hepatocyte-like cells. Regarding the P-glycoprotein (P-gp) efflux protein, its presence and function were predominantly confirmed in the control sample. This aligns with the expectation that P-gp is primarily located in cholangiocytes in the canine liver [59]. Consequently, this suggests that the control cell lines were mainly composed of functional cholangiocytes. This was further confirmed in experiments assessing P-gp transport activity. Both the differentiated organoids and the controls demonstrated functional P-gp. Moreover, the addition of Verapamil effectively inhibited P-gp-mediated efflux of rhodamine into the lumen of the hepatic organoids, confirming the functional activity of this efflux protein.

An interesting comparison of our data can be made with the findings in the study of Kruitwagen et al. [13], who used protocols for canine liver organoid culture and differentiation that had previously been developed by Nantasanti et al. [33]. Our M01 media composition (BMP7+DAPT + FGF19+Dex) was based on the original Nantasanti et al. report, however, the removal of Wnt described on Day 4 in the original protocol was not performed in our experiments as our organoid cells tended to undergo apoptosis within 2–3 days after Wnt withdrawal. Additionally, we did not remove FGF10 on day six as the original protocol suggests [13], as none of the other six protocols found in the literature described this step as critical for hepatocyte organoid culture [[8], [12], [24], [25],^14^].

While Nantasanti et al. reported measurable albumin production in the supernatant of their differentiated canine hepatic organoids, the absence of details regarding the length of culture for their tested media compositions makes direct comparisons with our results challenging. A similar issue arises when comparing our data to the albumin production levels reported by Huch et al. [15], as their manuscript lacks information on the volume of media used for the final albumin measurements.

Additionally, we observed hepatocyte ballooning in our M25 samples, likely induced by the artificial stimulation of organoid differentiation through the introduction of pro-inflammatory, disease-like conditions via media components. We also noticed signs of lipid and glycogen accumulation within the hepatocyte-like cells in our cultures. The latter was confirmed through histological staining for glycogen using PAS staining. These observations provide further insights into the complex morphological and functional changes that occur during hepatocyte differentiation in organoid cultures.

There were several limitations of our study. First, the number of organoid cell lines used in the experiments was limited. The original measures of albumin production in the culture supernatant were performed with one canine organoid hepatic cell line (in triplicates) due to technical constraints of culturing 24 different media simultaneously. Further experiments were performed on two canine organoid cell lines using 12 replicates. Additionally, cleaning of the cultures and lowering the seeding cell density were performed to limit the occurrence of apoptosis, and this approach could have influenced cellular differentiation, particularly by removing cells possibly undergoing epithelial-to-mesenchymal transition. Suspicion of lipid accumulation within the hepatocyte-like cells was not explored in our current study using paraffin-embedded samples. To obtain a more definitive identification of lipid accumulation, future experiments should employ appropriate methodologies, such as using frozen tissue slides designed for lipid staining. This approach would provide a more robust confirmation of lipid presence and its potential implications in the differentiation process. Additionally, a limitation of this study is the lack of primary hepatocyte use for these experiments. This kind of cell line would be useful to compare properties of our derived organoids to the primary cell culture. These experiments were omitted as they require the sacrifice of the animal. While some metabolic assays were incorporated into this experiment, our efforts would be better characterized if we included more (for example a Cytochrome P450 activity measurements). Unfortunately, we saw a problem in translation of human metabolic assays into canine population. Further investigation of organoids’ metabolic activity is therefore warranted.

The live cell counts in cultures incubated in media M16 and M11 were two and three times higher, respectively, than in control samples. Notably, media M01, which contained BMP7, DAPT, FGF19, and Dex, yielded a live cell count that was 15 times higher than the control group. Functionally, the organoids derived from these media compositions demonstrated enhanced albumin secretion. Morphologically, there was a partial differentiation into hepatocytes, although the percentage of mature hepatocytes (10%) was considerably lower than what is typically observed *in vivo* [60]. These findings suggest that while our media compositions were effective in promoting hepatocyte differentiation to some extent, further optimization is necessary to replicate the *in vivo* cellular landscape more closely. Organoid cell cultures that had been treated with M01 media further demonstrated that the FGF19 component in the media strongly supports the culture of ASC-derived hepatocytes by reducing the likelihood of apoptosis and promoting overall cell proliferation. FGF19 was not found to support hepatocyte differentiation in our experimental media compositions. Finally, our results highlight the necessity of removing R-spondin to allow proper hepatocyte differentiation in the canine organoid system. Future experiments using Fluorescence-activated cell sorting (FACS) for hepatocyte-like cells and subsequent organoid culture may further enhance the differentiation of adult hepatic stem cells towards a physiologically relevant 3d-cell culture exhibiting fully mature hepatocyte function.

## 5 Conclusion

In summary, the inclusion of HGF and FGF10 in our culture media compositions, along with the removal of ROCK inhibitor, CHIR99021, and Nicotinamide, was designed to promote hepatocyte differentiation. We report the successful use of specific culture media containing DAPT and Dex, which resulted in optimal conditions for improved function of canine hepatocyte-like cells in organoid cultures. BMP7 seemed to be important to facilitate hepatocyte differentiation, while FGF19 only played a supporting role in enhancing anti-apoptotic and proliferative functions in specific media compositions. Importantly, removal of R-spondin was essential for hepatocyte differentiation in our experiments.

## Data availability statement

Data is available upon request.

## CRediT authorship contribution statement

**Vojtech Gabriel:** Writing – review & editing, Writing – original draft, Visualization, Validation, Supervision, Software, Resources, Project administration, Methodology, Investigation, Formal analysis, Data curation, Conceptualization. **Addison Lincoln:** Writing – review & editing, Writing – original draft, Visualization, Validation, Resources, Methodology, Investigation, Formal analysis, Data curation, Conceptualization. **Christopher Zdyrski:** Writing – review & editing, Visualization, Validation, Software, Resources, Methodology, Investigation, Conceptualization. **Abigail Ralston:** Writing – review & editing, Resources, Methodology, Investigation. **Hannah Wickham:** Writing – review & editing, Validation, Methodology, Investigation, Conceptualization. **Sydney Honold:** Writing – review & editing, Visualization, Methodology, Investigation. **Basant H. Ahmed:** Writing – review & editing, Visualization, Validation, Methodology, Investigation. **Karel Paukner:** Writing – review & editing, Writing – original draft, Resources, Methodology, Investigation. **Ryan Feauto:** Writing – review & editing, Methodology, Investigation. **Maria M. Merodio:** Writing – review & editing, Validation, Resources, Conceptualization. **Pablo Piñeyro:** Writing – review & editing, Resources, Methodology, Investigation, Formal analysis, Data curation, Conceptualization. **David Meyerholz:** Writing – review & editing, Visualization, Supervision, Resources, Project administration, Methodology, Investigation, Formal analysis, Conceptualization. **Karin Allenspach:** Writing – review & editing, Writing – original draft, Supervision, Resources, Project administration, Methodology, Investigation, Funding acquisition, Formal analysis, Conceptualization. **Jonathan P. Mochel:** Writing – review & editing, Writing – original draft, Supervision, Resources, Project administration, Methodology, Investigation, Funding acquisition, Formal analysis, Data curation, Conceptualization.

## Declaration of competing interest

The authors declare that they have no known competing financial interests or personal relationships that could have appeared to influence the work reported in this paper.
